# Proserpine Again

**Published:** 1908-12

**Authors:** Eloise Shepherd

**Affiliations:** R. F. D. No. 3, Box 61A, Houston, Texas. Alias Proserpine


### Communications.

### Proserpine Again.

**Open Letter From a Talented Woman on a Prolific Cause of Insanity. An Introductory to Her Critique of Dr. Malchow's Book, "Sexual Life," to Appear in Next Issue of This Journal.***

The October issue of Texas Medical Journal contains an editorial relating to myself, of which I was not aware until the last day of October. I had requested Dr. Daniel to publish a review on Dr. C. W. Malchow's work entitled, "The Sexual Life." My request was accompanied by a statement to the effect that I had offered it to three different medical publications, and it had been returned with splendid excuses by each editor. I stated to Dr. Daniel that I was keeping these excuses, intending to publish them myself, as a sort of appendix to the review, which I purposed

*This letter was intended for the November number and announced in the October number, but was not received in time for publication.

issuing in small pamphlet form; and if he wished his excuse to appear with the other three, he might forward it when he returned the MS. He wrote me, agreeing to publish it in the November number, provided I allowed him the privilege of adding my letter as an introduction, and a few biographical extracts from my former article. Now, the "former article" was given to the public in the April number under the authorship of "Proserpine" (Mrs. Pluto). I am not unknown as a writer, in several States. I was superintendent of Social Purity, a department of the W. C. T. U., in Kansas before coming to Texas over twelve years ago. When I wrote that letter to Dr. Worsham last April I did not feel quite ready to appear publicly in the character of "Proserpine," though all the facts as related to him are true, and can be substantiated by me; the fact that, as stated by Dr. Daniel, "some of it is unprintable even in a medical journal," caused me to feel the shame a pure woman must ever be sensible of, wife though she be, when compelled to do things too vile to be spoken in print. This explanation constitutes my reason for using the *nom de plume* I did on that occasion. Intelligences with greater power than I, it seems, decreed that I must "face the music" anyway; hence I wrote both Dr. Worsham and Dr. Daniel at once, giving my name and assuring them I was abundantly able to support any statement I had made; and I can add to that now by saying, I can throw a great deal of additional light on the proposition I made in April, relative to depraved sex-life being the cause, directly and indirectly, of 99 per cent of the insanity now afflicting the race. I am not only a mother and a grandmother, but I am a great grandmother also; and my lessons in life qualify me to "speak with authority." I know too many instances where healthy, bright, happy young girls, having contracted matrimonial relations, were reduced to conditions so pitiable in a few short years—or a few months—as to indicate all too clearly the awful cause; especially when taken in connection with their recital of usages to which they had been subjected. That the same affliction curses men, also, but emphasizes the dreadful truth of degraded humanity, and a prodigal waste of life's most sacred forces.

It is a fact that a larger number of women than men are sufferers from neurotic ailments. Why? The instrument from which can be evolved the finest tones is most easily unstrung by fiercest forces falling upon its fineness; and woman is conceded to be the finer human instrumentality. Being the finer organism, she needs the finer forms of loving expression. Allow her to discern that no single caress, no tender affection or endearment is ever offered her except with the accompaniment of a sex demand, and how can she do else than distrust the genuineness of the professed love? A fine horse is valued for his service. His owner is kind and good to him; and urged, perhaps, by a type of appreciation, he occasionally offers a little patting or expression of kindliness. Endow the animal with consciousness, such as a human soul is supposed to be, and he would know that all his master cared for him was to ride or drive, or in some way enjoy his service. Has man no clearer intuitions than to understand that when a woman is appreciated only for whatever of gratification she can render him, she can not sustain the love-fires that prompted her to matrimonial union.

Repeating that I speak with authority—meaning the experiences of numerous sister women additional to my own—I want to emphasize this one and most vital point: that a woman finds an exceedingly high degree of pure joy in simply giving a love she feels in her heart. The privilege of fondling and kissing and showing forth her deep love-nature in the purely affectional manner native to woman, yields her the most exquisite happiness; but if she never dare reach forth her arms to embrace her husband, nor show him the sweet tenderness of her heart except she raise a hurricane of lust which must find exercise upon her already surfeited body, she soon grows to fear permitting herself the delight of giving, though she do not receive, the endearments her heart-self craves so much. The same fervency of action that precedes the sex impulse, if an occasionally flowing from the heart, would invoke the very needs the husband most desires; but failing to receive the cherishing, the life-giving proofs of a real love for the wife, given by the husband, her love-nature dies out. Then her body, still subject to devastating demands from which her whole soul turns with indescribable loathing, as a sick stomach from the cause of its revulsion, she grows neurotic; is subject to mental as well as physical decay; and either the kindly grave or the awful asylum hides forever the poor victim of "too much hawk." If the truth is ever to be unveiled, if the victim's side is ever to be spoken, lips above ground and outside asylum walls must do it. Personally, I have nothing to lose by giving forth these facts; nor, be it remembered, have I aught to fear. Let the world of men challenge me from any plane or walk, and they will speedily find me provided with facts that will no more "down" than did Banquo's ghost; for whatever can be supported by statistics will scarcely yield to "hush, hush, those things are not to be talked about." I repeat my query: Why is it right to do what dare not be told or written? Were these things not done, they could not be told or spoken. Degrading the very fountain of life, the handiwork of the Infinite to a plane too shameful for human speech! It would seem He would strike dumb the creatures who so defiled that wondrous thing, creative power, when it had been so shamed as to be unfit for utterance by that other divine thing also a gift of the Maker—wonderful, wonderful speech!

I find a deep, deep, unalloyed happiness in just loving. To stroke the arched neck of a noble horse; pat the head of an intelligent dog, in whose eyes shines an almost human intelligence; or gently pet a lovely kitten or bird; all these things yield me exquisite happiness. How much deeper and higher and more infinitely joyous would be the privilege of lovingly associating with a kindred soul of the masculine sex, were it possible to do so on the equally safe plane as with the lower animals. I am not advertising for companionship in this respect. Three trials are abundantly sufficient for me. I am devoting my energies to other channels of action which thus far have borne me a much greater share of joy in existence. So far as my knowledge goes and the deductions I am forced to make from it, dogs and horses are more desirable associates for me. Let other people decide the question for themselves. I am at liberty to act for myself.

Women under the rule of Christian civilization are less free than the cattle on the plains. The female of the brute creation has no need of fear that she or her young will suffer for food or shelter except she accede to male demands at his behest and not her own. In that sense the human female is living on a scale far below that of the brute female. Nor could the brute female ever engender a natural sex impulse were she made to constantly serve the needs of the male, nor allowed time to conserve her own forces for sacred use in the sphere of procreation, or the begetting of her kind.

I am mailing a communication to *McClure's Magazine* entitled, "The Psychology of Drunkenness." They may not accept it; but if not, some other publication will, and possibly it may be the TEXAS MEDICAL JOURNAL. Meantime, my review of Dr. Malchow's "The Sexual Life," will most certainly appear in the January number. If creation is of God, it must be holy and righteous; and procreation being of man, made in the image, and claiming to be a son of God, it, too, must be holy and righteous. Then why and how has it come to be a matter so vile that it dare not be spoken or written about? Please, someone, answer. Meantime, let me tell a dream I had many years ago, ere my knowledge of sin and sorrow had become so wide. This dream was during my early girlhood. I had been taught of God and heavenly things at Sunday school and church; and in my primitive experiences had formed a tolerably definite idea of what heaven would be like. I had given no thought of the nature of hell, not expecting to go there. I had no interest in the place. I dreamed I died, and, of course, was sailed away to heaven. The arrangement of plants and flowers and general scenes, the trees of life and rivers of delight all corresponded to my idealization gained on earth. I had seen pictures of thrones, and was not surprised to find one there. On it sat three Supreme Beings, instead of one, however; the center one larger than the other two. When I appeared for judgment, one of them opened a kind of big book case and took down a huge volume, measuring about four by six feet. They commenced looking through it, but seemed not to find what they looked for, and replacing it, got another. They having gone about through it, I observed their countenances were growing solemn in expression. Finally, closing the book, they announced that I had not been good enough while on earth to be allowed a dwelling place in heaven, so I must prepare to depart. How I reached the nether place, I am not sensible; but I seemed to "take a tumble" somehow, and found myself in the region called hell. Remember, I had never pictured it in my earthly meditations, hence was greatly surprised at what I saw. Description of it is not easy; but as nearly as I can convey an idea, it was a pit dug into the ground—if ground was there—measuring perhaps three hundred feet in diameter. It was filled with molten fire to within about fifty feet of the top. This molten fire was partly composed of living beings, all writhing and squirming like live eels in and through each other; eyes, hands, limbs and all resembling red hot iron. Across the surface were extended beams or girders or whatever term best symbols the "gridiron" that covered the pit. It was a part of the sentence of each soul to sit on this gridiron for a probationary period, and at the expiration of the time decreed, descend into the burning lake also. I took my place there to await my descent. Occasionally I noticed, now here, now there, one whose time expired, and down they dropped; I knowing my time was arriving.

Around the sides of this "hades" and builded into the walls about half way between the fire and the ground surface were iron doors about three by four feet, blackened with smoke, and, in a way, resembling the furnace doors of locomotives. I knew, with the strange intuitional powers the soul seems given when freed from bodily sensibility, that inside these doorways the devils dwelt; for inasmuch as there were more Gods than one, so it seemed there were more devils than one, also. While I sat biding my time, one door, rather larger and more ornamental than the rest, and in which I instinctively knew dwelt the master devil, opened and the king of the hades-world came out. He walked about leisurely, his anchored tail dropping down between the beams sometimes, and once or twice he stumbled; and I recall a feeling of fear lest he might fall in. At first he did not notice me at all; but in his round of inspection he spied me. His actions then I can not tell of better than to say I've seen such in public places, on trains, etc., when a man wished to attract the favorable notice of a woman. He edged toward me in a manner that indicated he was looking the other way. A glance once in a while directed toward me (I was watching, it will be noted, had little else to do) and finally, after pretending to be looking at some of the other condemned ones very carefully, happened (?) to move quite close to me. When he was near enough to speak he asked why I was there. I told him I did not know; I had always tried to be a good girl on earth, and had no intimation of why the universal judge had sent me to his dominions. *He was in sympathy with my case at once.* He said he did not know why he had been cast out of heaven, either; that he had only done the things considered most divine as men understand divinity. I remember we discussed the subject at some length, and the climax of my dream, I'll wager, not one man in ten thousand would guess! He invited me to go home and live with him; and then I would be saved from the fire below. Being in a position where choice was limited, I accepted his proposal; although his features, long teeth, cloven feet, donkey ears and general appearance would scarcely attract me now. It may be worthy of reflection that my *nom de plume*, selected for my communication in writing to Dr. Worsham last April, was not wholly inappropriate, though I did not think of my early dream at the time.

The fact, however, the shining truth, the consoling feature of my experience in hades, remains as yet untold. He did not offer the least suggestion of a sexual nature in my housekeeping experience with him—the devil—and through a "right ascension" mental view the hadesiacal trip and visit I enjoyed at that time. His majesty grows in my esteem daily. I awoke soon after reaching the house, however, and don't believe there was any night there.

This is a dessert on which to subsist till the next issue of the Texas Medical Journal, when the promised review will be forthcoming.

Mrs. Eloise Shepherd, B. O.,
R. F. D. No. 3, Box 61A, Houston, Texas.
Alias Proserpine.

